# Bilateral primary psoas abscesses due to methicillin-resistant *Staphylococcus aureus* in a neutropenic patient: a case report

**DOI:** 10.1186/s13256-015-0781-7

**Published:** 2016-01-19

**Authors:** Faris G. Bakri, Azmy M. Hadidy, Fadi Hadidi, Nosaiba Ryalat, Lina Saket, Nour Shurbasi, Jamal Melhem

**Affiliations:** 1 Department of Medicine, Division of Infectious Diseases, The University of Jordan, Jordan University Hospital, Amman, 11942 Jordan; 2 Department of Radiology Medicine, The University of Jordan, Jordan University Hospital, Amman, Jordan; 3 Division of Orthopedic Surgery, The University of Jordan, Jordan University Hospital, Amman, Jordan; 4 Department of General Surgery, The University of Jordan, Jordan University Hospital, Amman, Jordan

**Keywords:** Methicillin-resistant *Staphylococcus aureus*, Neutropenic fever, Percutaneous drainage, Psoas abscess

## Abstract

**Background:**

Pyogenic abscess of psoas muscles is a rare condition. Psoas abscess due to methicillin-resistant *Staphylococcus aureus* is an emerging and rare infection and so far the related data are scarce.

**Case presentation:**

We report the rare case of primary and bilateral large psoas abscesses due to methicillin-resistant *Staphylococcus aureus* in a 54-year-old Arab Jordanian woman with breast cancer who had neutropenia after starting chemotherapy. She was diagnosed 50 days after onset of symptoms. However, despite this delay in diagnosis and the large size of the abscesses, she had a full recovery. She was treated with antibiotics and percutaneous drainage and was doing very well at a follow up of 18 months.

**Conclusions:**

Psoas abscess due to methicillin-resistant *Staphylococcus aureus* might have insidious presentation with extensive disease especially in immunocompromised patients. However, it can be managed effectively with percutaneous catheter drainage and appropriate antibiotic therapy.

## Background

Pyogenic abscess of psoas muscles is a rare condition with a mortality rate of 100 % if left untreated [1]. The actual incidence is not known but is believed to have increased in recent years due to improvements in imaging technology. In one study [2], a significant statistical increase in rates from 0.5 cases per 10,000 hospital admissions from 1993 to 2004 to 6.5 cases per 10,000 hospital admissions from 2004 to 2007 was reported. Nonetheless, the condition is still regarded as a rare disease even in recent studies [3].

Although *Staphylococcus aureus* is the leading cause for primary psoas abscess, psoas abscesses due to methicillin-resistant *Staphylococcus aureus* (MRSA) have been rare until recently when increasing rates have been reported in the last few years [2, 4, 5]. Therefore, data on psoas abscess due to MRSA are lacking especially in regard of treatment [2]. Here, we report a rare case of primary and bilateral large psoas abscesses due to MRSA in a neutropenic patient who eventually had an excellent outcome.

## Case presentation

A 54-year-old Arab Jordanian woman who had a history of breast cancer 11 years prior to her current complaint, presented in 2012 with a left breast mass due to recurrence of her cancer. The mass of the recurrent cancer was excised and chemotherapy with docetaxel, doxorubicin, and cyclophosphamide was started. However, in October 2012 and 7 days after the second cycle, she was admitted because of fever, chills, and right lower quadrant pain of 3 days’ duration. Her physical examination showed epigastric and right lower quadrant tenderness and her white blood cell count (WBC) was 1.28×10^9^/L (neutrophils 10 %). Therefore, she was treated with imipenem with moderate improvement and was discharged after 4 days on cefixime orally.

Later, and after the third cycle of chemotherapy which consisted of docetaxel and cyclophosphamide, she was admitted again for fever. Her laboratory tests were unremarkable except for hemoglobin of 7.9 g/dL, WBC 2.6×10^9^/L (neutrophils 15 %), and platelets of 161×10^9^/L. During her stay, however, she complained of left hip and knee pain. Her examination showed tenderness over her great trochanter with full range of movement. Therefore, a dedicated left hip magnetic resonance imaging (MRI), without pelvic cuts, was performed and was reported as normal. Her blood and urine cultures remained negative. She was treated with imipenem and vancomycin and was later discharged after 8 days of hospital stay.

**Fig. 1:**
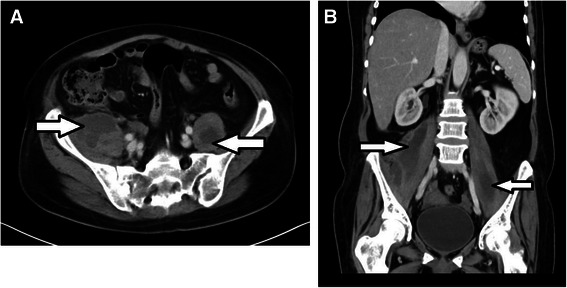
Axial (**a**) and coronal (**b**) abdomen and pelvis computed tomography scan with intravenous contrast showing bilateral psoas muscle abscesses (*arrows*)

In December 2012, before receiving the fourth cycle of chemotherapy, she developed fever, chills, and night sweats. Her physical examination showed that she was ill with temperature of 38.7 °C and pulse rate of 90/minute and her laboratory tests showed WBC of 13.5×10^9^/L (neutrophils 73 %). An abdomen computed tomography (CT) was subsequently performed, 50 days after onset of symptoms, and showed large bilateral psoas abscesses: the right measuring 13×8×5 cm and the left 9×5×3.5 cm (Fig. 1). Therefore, a bilateral CT-guided percutaneous drainage (PCD) was inserted. Culture from the pus grew MRSA which was sensitive to vancomycin, gentamicin, tigecycline, levofloxacin, erythromycin, clindamycin, and chloramphenicol by disk diffusion test. A lumbosacral MRI with pre- and post-contrast sagittal sequences showed no evidence of spondylodiscitis. A transthoracic echocardiogram was normal. She denied unsafe sexual practice, recent travel, trauma, alcohol, or recreational drug use.

After 24 hours of drainage, she became afebrile, was able to move her hip without pain, and had dramatic improvement. She was treated with vancomycin 1 gm administered intravenously every 12 hours for 1 week which was then switched to teicoplanin 400 mg administered intramuscularly once daily as an out-patient for another 3 weeks. One week after drainage, the catheters were removed and she went home. She received her fourth cycle of chemotherapy (docetaxel and cyclophosphamide) after 4 weeks of drainage without complications and later on completed another three cycles of chemotherapy. At follow up of 18 months, she was doing well and had no evidence of recurrence of infection on repeat CT imaging.

## Discussion

Psoas abscess is classified as either primary or secondary. A primary abscess occurs due to the hematogenous or lymphatic spread of the causative organism from a distant site. Secondary abscess occurs as a result of the direct expansion of a nearby infectious or inflammatory process into the psoas muscle [6]. Primary abscess occurs most commonly in patients with a history of diabetes, injection drug use, alcoholism, acquired immune deficiency syndrome, renal failure, hematological malignancies, immunosuppression, or malnutrition [7]. Primary abscesses account for 99 % of abscesses in Asia and Africa while in Europe and North America the prevalence is lower with rates of 18 % and 61 % respectively [1, 6, 7].

Our case has several rare and interesting features: the primary type, the infection with MRSA, the bilateral location, the large size of the abscess, the immunocompromised host due to chemotherapy and neutropenia, and the excellent outcome of the patient.

Bilateral psoas abscesses are rare; Ricci *et al*. [1] in a large case series that included every case published in the literature until 1986 found a rate of 1.4 % of bilateral abscesses among cases of primary or secondary types. Navarro López *et al*. [8] in a comprehensive retrospective case series which consisted of 124 cases from 11 hospitals in Spain found a rate of 4 % of bilateral abscesses. Alonso *et al*. [2], in a case series of 61 patients from 1993 to 2007, found a rate of 16 % of bilateral abscesses. Here, the bilateral location and the large size of the abscesses are probably due the late diagnosis, the immunocompromised host, and the virulence of the infecting pathogen.

The predominant organism in primary psoas abscess is *S. aureus*; Ricci *et al*. [1], in their large record of case series up to the year 1986, found that *S. aureus* caused 88 % of cases followed by *Streptococcus* (5 %) and *Escherichia coli* (3 %). Lai *et al*. [3] in another record of case series from 1986 to 2011 also found that *S. aureus* was still the dominant etiology. However, despite the fact that *S. aureus* was the predominant etiology, MRSA remained a rare etiology until recently [4, 8]. Two reports have recently documented increasing infection with MRSA: Alonso *et al*. [2] and Kim *et al*. [5]. Alonso *et al*. [2] reported the emergence of MRSA as an increasingly common pathogen in a series of 61 cases from a tertiary care center in the USA. Kim *et al*. [5] also showed an increase in infection with MRSA, in Korea, among 75 cases between two periods: (2001–2006) , and (2007–2012). Other recent published cases series found rates of infection with MRSA of two out of 42 cases (4.8 %) [9] and 10 out of 88 (11.4 %) [10].

The phenotype of the isolated MRSA here (sensitive to levofloxacin, erythromycin, and clindamycin) suggests it is a community-associated MRSA. However, this phenotype criterion remains inaccurate and nucleic acid amplification methods to detect *mecA* would have been more sensitive to differentiate between community and health care MRSA [11]. Since we did not perform any molecular tests here, we had to rely on clinical criteria alone to classify the infection as health care-associated infection because it presented after admission to our hospital. Of note, data from our hospital and other hospitals in Jordan indicate high prevalence of MRSA including strains with intermediate resistance to vancomycin [12, 13]. We initially opted to treat the patient with vancomycin because it is the drug of choice in such severe infections [11]. Later, we switched her therapy to teicoplanin because of the easier intramuscular administration in the out-patient setting and the similar efficacy to vancomycin [14].

Currently, whether PCD or surgical intervention should be used in patients with psoas abscess remains controversial because few studies with large sample sizes have been performed [10]. However, at present, PCD is considered preferable to surgical intervention for the treatment of psoas abscess [3, 10]. Recently, Hsieh *et al*. [10] in a retrospective series of 88 cases, found that either PCD or primary surgical intervention is a suitable treatment for patients with non-gas-forming psoas abscess. Catheter dislocation and obstruction lead to the majority of complications. However, overall low mortality and morbidity rates are major advantages of CT-guided drainage as it is a safe, effective, and less invasive treatment [15].

## Conclusions

MRSA is an increasingly reported and emerging etiology in psoas abscess. Our case has several rare and interesting features specifically: the primary type, the infection with MRSA, the bilateral location, the large size of the abscesses, the immunocompromised host, and the excellent treatment outcome. Psoas abscess is an important condition in the differential diagnosis of patients with low back and hip pain. MRI or CT scan could establish the diagnosis and define the extent of the psoas abscess. Percutaneous catheter drainage with appropriate antibiotic therapy can be effectively used in management.

## Consent

Written informed consent was obtained from the patient for publication of this case report and any accompanying images. A copy of the written consent is available for review by the Editor-in-Chief of this journal.

## Abbreviations

CT: Computed tomography
MRI: Magnetic resonance imaging
MRSA: Methicillin-resistant *Staphylococcus aureus*
PCD: Percutaneous drainage
WBC: White blood cell count
